# Proteomic Profiling of BRAFV600E Mutant Colon Cancer Cells Reveals the Involvement of Nucleophosmin/c-Myc Axis in Modulating the Response and Resistance to BRAF Inhibition by Vemurafenib

**DOI:** 10.3390/ijms22126174

**Published:** 2021-06-08

**Authors:** Petra Grbčić, Dora Fučkar Čupić, Tania Gamberi, Sandra Kraljević Pavelić, Mirela Sedić

**Affiliations:** 1 Department of Biotechnology, University of Rijeka, Radmile Matejčić 2, 51000 Rijeka, Croatia; petra.grbcic@biotech.uniri.hr; 2 Faculty of Medicine, University of Rijeka, Ul. Braće Branchetta 20/1, 51000 Rijeka, Croatia; dorica9@gmail.com; 3 Dipartimento di Scienze Biomediche, Sperimentali e Cliniche Mario Serio, University of Florence, Viale Morgagni 50, 50134 Florence, Italy; tania.gamberi@unifi.it; 4 Faculty of Health Studies, University of Rijeka, Viktora Cara Emina 5, 51000 Rijeka, Croatia; sandrakp@uniri.hr

**Keywords:** colorectal cancer, BRAFV600E mutation, vemurafenib, PLX4032, chemoresistance, nucleophosmin, c-Myc, proteomic, bioinformatics

## Abstract

BRAFV600E mutations are found in approximately 10% of colorectal cancer patients and are associated with worse prognosis and poor outcomes with systemic therapies. The aim of this study was to identify novel druggable features of BRAFV600E-mutated colon cancer (CC) cells associated with the response and resistance to BRAFV600E inhibitor vemurafenib. Towards this aim, we carried out global proteomic profiling of BRAFV600E mutant vs. KRAS mutant/BRAF wild-type and double wild-type KRAS/BRAF CC cells followed by bioinformatics analyses. Validation of selected proteomic features was performed by immunohistochemistry and in silico using the TCGA database. We reveal an increased abundance and activity of nucleophosmin (NPM1) in BRAFV600E-mutated CC in vitro, in silico and in tumor tissues from colon adenocarcinoma patients and demonstrate the roles of NPM1 and its interaction partner c-Myc in conveying the resistance to vemurafenib. Pharmacological inhibition of NPM1 effectively restored the sensitivity of vemurafenib-resistant BRAF-mutated CC cells by down-regulating c-Myc expression and activity and consequently suppressing its transcriptional targets RanBP1 and phosphoserine phosphatase that regulate centrosome duplication and serine biosynthesis, respectively. Altogether, findings from this study suggest that the NPM1/c-Myc axis could represent a promising therapeutic target to thwart resistance to vemurafenib in BRAF-mutated CC.

## 1. Introduction

BRAFV600E mutations are found in approximately 10% of colorectal cancer (CRC) patients and are associated with sustained cell proliferation, diminished apoptosis and acquired resistance to standard and targeted chemotherapies [1]. In CRC, the BRAFV600E mutation confers worse prognosis, decreased overall survival and poor outcomes with systemic therapies. In general, BRAFV600E mutant CRC exerts distinctive molecular, pathological and clinical features. Specifically, the BRAFV600E mutation is commonly associated with the serrated adenoma pathway, right-sided CRC, T4 stage, poor differentiation and mucinous histology and is prevalent in elderly female patients [2]. In addition, tumors bearing a BRAF mutation often show microsatellite instability (MSI), hypermethylation, higher incidence of CpG island methylation and distinct patterns of metastasis characterized mainly by peritoneal and nodal metastases and, less likely, lung metastases.

Although treatment with single-agent BRAFV600E inhibitor vemurafenib (PLX4032) resulted in promising response rates in metastatic melanoma patients harboring the BRAFV600E mutation, clinically meaningful activity was not achieved in BRAF mutant metastatic CRC patients, which implies that the BRAFV600E-regulated response and resistance to therapy mirror tissue- and cancer-type specific biological contexts defined by the BRAFV600E mutation. The heterogeneous clinical response to targeted therapies observed in BRAFV600E mutant CRC patients has spurred investigation into the molecular basis underlying these differences, which has led to the discovery of two gene expression-based subgroups of BRAFV600E-mutated CRC, one exerting KRAS/AKT pathway activation, mTOR/4EBP deregulation and epithelial–mesenchymal transition (EMT), and the other showing dysregulation of the cell cycle [3]. Expectedly, the same study indicated that these two subtypes have different sensitivities in silico to targeted chemotherapy drugs including BRAF and MEK inhibitors, reflecting their observed molecular differences.

The functional consequences of the BRAFV600E mutation in CRC and its impact on molecular processes that govern cell behavior and response to chemotherapy have not been fully elucidated. Several efforts have been made to investigate the underlying biological context of the BRAFV600E mutation in CRC by capturing specific changes in the gene expression levels that could distinguish tumors bearing the BRAFV600E mutation from other oncogenic mutations and molecular types of CRC [4,5,6,7]. The distinctive gene expression signature of BRAFV600E mutant CRC could serve as a classification and prognostic tool and could also reveal novel therapeutic vulnerabilities of BRAF mutant CRC, which would enable the development of tailored treatment for CRC patients with the BRAFV600E mutation. Based on the genes that were specifically up-regulated in BRAF mutant CRC in comparison with BRAF and KRAS wild-type CRC, Vecchione et al. [6] were able to identify spindle formation and mitotic progression as distinct druggable features of BRAFV600E mutant colon cancer cells and revealed the ability of vinorelbine, a vinca alkaloid that prevents formation of the mitotic spindle, to selectively suppress the growth of BRAF-mutated colon cancer cells both in vitro and in vivo. Another example showing the utility of the BRAFV600E mutant-specific gene expression signature as a target for novel candidate therapy in CRC was provided by San Lucas et al. [8], who employed an in silico approach to reveal specific gene expression signatures of BRAFV600E mutant CRC as compared to BRAF wild-type CRC and identified EGFR inhibitor gefitinib and proteasome inhibitor MG-262 as potential candidate drugs to specifically treat BRAFV600E CRC.

In the present study, we set out to explore global proteomic features of BRAFV600E mutant colon cancer cells in comparison with KRAS mutant/BRAF wild-type and double wild-type KRAS/BRAF colon cancer cells in order to identify novel druggable vulnerabilities of BRAF-mutated colon cancer that could play a role in regulating the cell response and resistance to BRAFV600E inhibition by vemurafenib. Bioinformatics analysis of proteins specifically up-regulated in BRAF-mutated cells revealed several cellular events that could be potentially associated with the BRAFV600E mutation in colon cancer cells including cytoskeleton remodeling, cell–cell adhesion, transcriptional and epigenetic dysregulations, increased ribosome biogenesis and protein synthesis, positive regulation of the cell cycle, centrosome cycle and mitosis, mitochondrial biogenesis and metabolic alterations linked with increased activity of the enzymes regulating methionine and serine biosynthesis. Importantly, our study reveals an increased abundance and activity of nucleophosmin (NPM1) that could be linked with initiation of centrosome amplification and mitosis as a novel molecular trait of BRAFV600E-mutated colon cancer in vitro, in silico and in tumor tissues from colon adenocarcinoma patients and provides evidence to demonstrate the roles of NPM1 and its interaction partner c-Myc in conveying the resistance to BRAF inhibitor vemurafenib. Pharmacological inhibition of NPM1 was proved effective in restoring the sensitivity of vemurafenib-resistant colon cancer cells with the BRAFV600E mutation by mechanisms that include down-regulation of c-Myc expression and activity and consequent suppression of its transcriptional targets RanBP1 and phosphoserine phosphatase regulating centrosome duplication and serine biosynthesis, respectively. Collectively, findings from this study provide ample indication that the NPM1/c-Myc axis could represent a promising therapeutic target to thwart resistance to vemurafenib in BRAF-mutated colon cancer.

## 2. Results

### 2.1. Proteomic Profiling of Colon Cancer Cell Lines Differing in BRAF Mutation Status

In order to identify proteomic features specifically associated with the BRAFV600E mutation in colon cancer and to exclude, at the same time, the possible interference resulting from the genetic diversity of different cell lines, we performed comparative proteomic profiling of the three selected colon cancer cell lines having the same MSI status, wt *PTEN*, wt *PIK3CA* and mutated *p53*, and differing only in BRAF/KRAS mutational status as follows: HT-29 (BRAFV600E/KRASwt), SW480 (BRAFwt/KRASG12V) and Caco-2 (BRAFwt/KRASwt).

**Figure 1:**
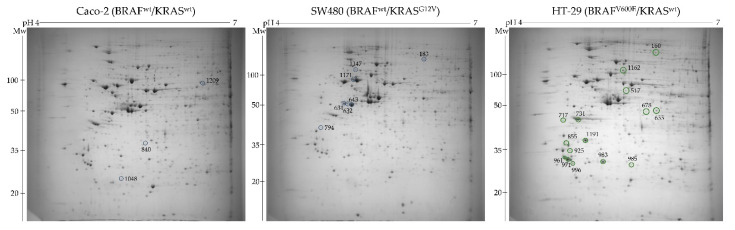
Representative 2-DE gel images of total cell proteome obtained in the pH range 4–7 from the three colon cancer cell lines differing in BRAF mutational status. Marked with a green circle are up-regulated protein spots detected in BRAFV600E mutant cells, while blue circles designate down-regulated protein spots.

Towards this aim, total cell proteins were resolved by two-dimensional polyacrylamide gel electrophoresis (2-DE) (Figure 1) followed by gel image analysis that enabled the detection of a total of 950 protein spots.

Among these, 166 spots were differentially expressed with a statistical significance (ANOVA *p*-value < 0.05) between the three cell lines, from which 16 and 10 were up- and down-regulated, respectively, specifically in the cell line harboring the BRAFV600E mutation in comparison to the cell lines carrying wild-type BRAF. These protein spots were manually excised, and their identity was revealed by MALDI-TOF/TOF mass spectrometry. Lists of differentially abundant protein spots between BRAF-mutated and BRAF WT cell lines are given in Table 1 and Table 2.

### 2.2. Bioinformatics Analysis of Proteomic Data

#### 2.2.1. Functional and Pathway Enrichment Analysis

In an attempt to better understand a molecular and cellular context in which the identified up-regulated proteins function, we performed bioinformatics analysis using several publicly available tools. First, we investigated biological functions of up-regulated proteins using the DAVID database for Gene Ontology functional annotation. The three GO categories, namely, cellular component (CC), biological process (BP) and molecular function (MF), were detected using DAVID. The top GO terms for differentially expressed proteins with increased abundance in the BRAFV600E cell line are shown in Supplementary Tables S1–S3.

The cellular component (CC) analysis revealed that up-regulated proteins were mostly localized to the intracellular part, cytoskeleton and adherens junction, as well as to the extracellular part (exosomes and vesicles) (Supplementary Table S1). For biological process (BP), up-regulated proteins were mainly enriched in organelle organization, regulation of cytoskeleton organization, mitochondrial membrane organization, cell–cell adhesion, positive regulation of the cell cycle (namely, G1/S phase transition) and centrosome cycle and intracellular transport including RNA export from the nucleus (Supplementary Table S2). Up-regulated proteins in the molecular function category were significantly associated with poly(A) RNA binding, cadherin binding and protein binding involved in cell–cell adhesion (Supplementary Table S3).

Next, we carried out pathway enrichment analysis based on the input from the up-regulated proteomic dataset using the Reactome database, where *p* < 0.05 was considered a threshold value. This analysis revealed 33 pathways with statistical significance in which up-regulated proteins seem to be involved (Table 3). The pathways related to the cell cycle, G2/M checkpoints, condensation of prometaphase chromosomes and G2/M DNA damage checkpoint are associated with the modulation of the mechanisms that regulate the cell cycle and progression through mitosis. Interestingly, Reactome analysis also showed significant enrichment for the pathway associated with epigenetic mechanisms. These include demethylation of histones by histone demethylases (HDMs) that generates formaldehyde, which is transferred to homocysteine to generate methionine, which is then cycled back to S-adenosylmethionine (SAM), a methyl group donor in many important transfer reactions including DNA methylation. SAM may also be used to regenerate methionine in the methionine salvage pathway, and the latter was also identified by Reactome analysis (Table 3). Another metabolic alteration potentially linked with the BRAFV600E mutation as revealed by Reactome analysis appears to include serine biosynthesis. 

Given that metabolism of serine and methionine is intimately linked with the activation of mTOR signaling [9,10], it is no surprise that the component of this signaling pathway, namely, mTORC1, which is the major effector of the PI3K-AKT pathway, also emerged from the Reactome analysis. mTOR has been previously identified as a potential therapeutic target in BRAFV600E-driven colorectal cancer [11]. In particular, the PI3K/mTOR pathway was shown to be one of the mechanisms underlying the resistance of BRAFV600E colon cancer cells to BRAF inhibition that could be overcome by concurrent BRAF and PI3K/mTOR blockade [12]. 

#### 2.2.2. PPI Network Construction and Module Analysis

In order to predict and analyze functional associations of proteins identified in the up-regulated dataset, we used the Search Tool for Retrieval of Interacting Genes (STRING) (https://string-db.org/, accessed on 27 May 2020) database that encompasses known and predicted protein–protein interactions (PPI) followed by identification and analysis of functional clusters in the PPI network. 

**Figure 2:**
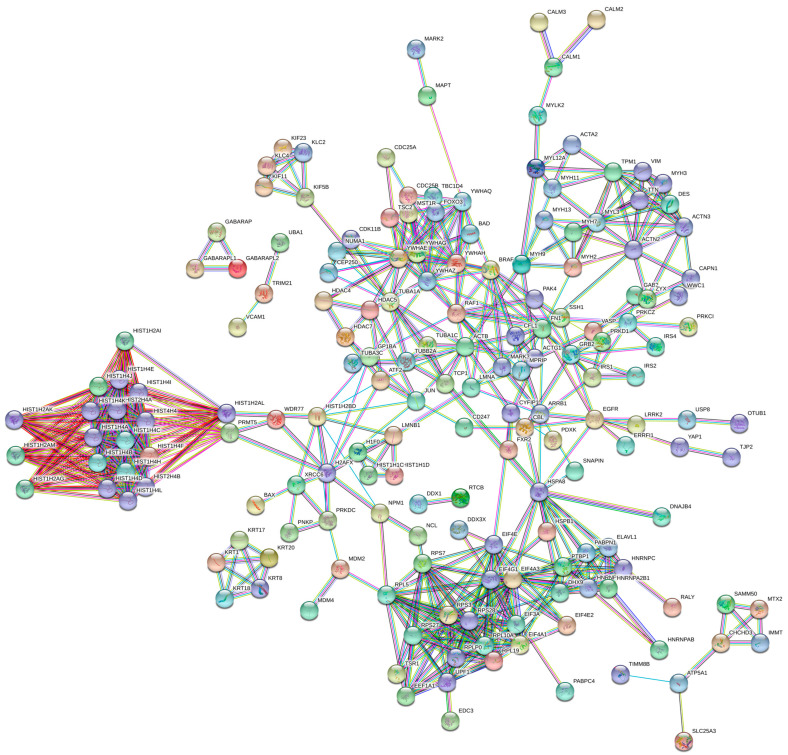
Protein–protein interaction (PPI) network of up-regulated proteins in BRAFV600E mutant HT-29 colon cancer cells and their interaction partners retrieved from the DAVID functional enrichment tool. The PPI network was constructed using the STRING database of known and predicted protein–protein interactions with the highest confidence of 0.900.

Starting initially from the list of differentially expressed proteins with a significantly increased abundance in the BRAF mutant colon cancer cell line, we retrieved the enriched protein–protein interaction dataset from the DAVID online functional annotation tool, which was then uploaded into STRING to finally construct the PPI network consisting of 235 nodes and 641 edges, with a confidence score of 0.900 and an average local clustering coefficient of 0.546 (PPI enrichment *p*-value < 1.0 × 10^−16^) (Figure 2).

We next visualized the network in Cytoscape, and using the MCODE plugin, we identified and visualized eight significant modules/clusters with an MCODE score of ≥4 and a number of nodes of >4 (Supplementary Table S4). Using the Cytohubba plugin, we then ranked the top 10 hub genes in each significant module based on two topological analysis methods: maximal clique centrality (MCC) and degree (Supplementary Table S4). Functional analysis of the genes in important modules revealed that they were mainly associated with histone structure and modifications (demethylation, methylation and deacetylation), pointing to epigenetic mechanisms (module 1); ribosome structure, biogenesis and protein synthesis (module 2); RNA metabolism and signaling by interleukins (module 3); actin cytoskeleton, cell movement, cell–cell communication and cell cycle regulation via G2/M checkpoints (module 4); regulation of microtubule dynamics and chromosome and centrosome positioning during mitosis (module 5); organization and structure of the keratin cytoskeleton (module 6); mitochondrial biogenesis (module 7); chromosome maintenance, nucleosome assembly, centromere organization and activation of transcription (module 8).

Altogether, bioinformatics analyses provided a broader perspective on the obtained proteomics data by revealing several events that might be associated with the BRAFV600E mutation in colon cancer including cytoskeleton remodeling, transcriptional and epigenetic dysregulations, increased ribosome biogenesis and protein synthesis, positive regulation of the cell cycle, centrosome cycle and mitosis and metabolic alterations linked with methionine and serine biosynthesis. 

### 2.3. In Vitro and In Silico Validation of Proteomic Data Reveals Increased Abundance of Nucleophosmin in BRAFV600E-Mutated Colon Cancer

Based on the observations from bioinformatics analyses, we opted for further in vitro validation of several protein targets arising from proteomic data that specifically regulate ribosome biogenesis, centrosome duplication and histone assembly (NPM1), mitotic spindle assembly and progress through mitosis (RanBP1), protein synthesis (eIF4E) and methionine (ENOPH1) and serine (PSPH) biosynthesis. Since many of these proteins including NPM1 [13], RanBP1 [14], eIF4E [15] and PSPH [16] were previously reported to be transcriptional targets of c-Myc, expression of c-Myc in colon cancer cell lines differing in their BRAF mutational status was also examined.

**Figure 3:**
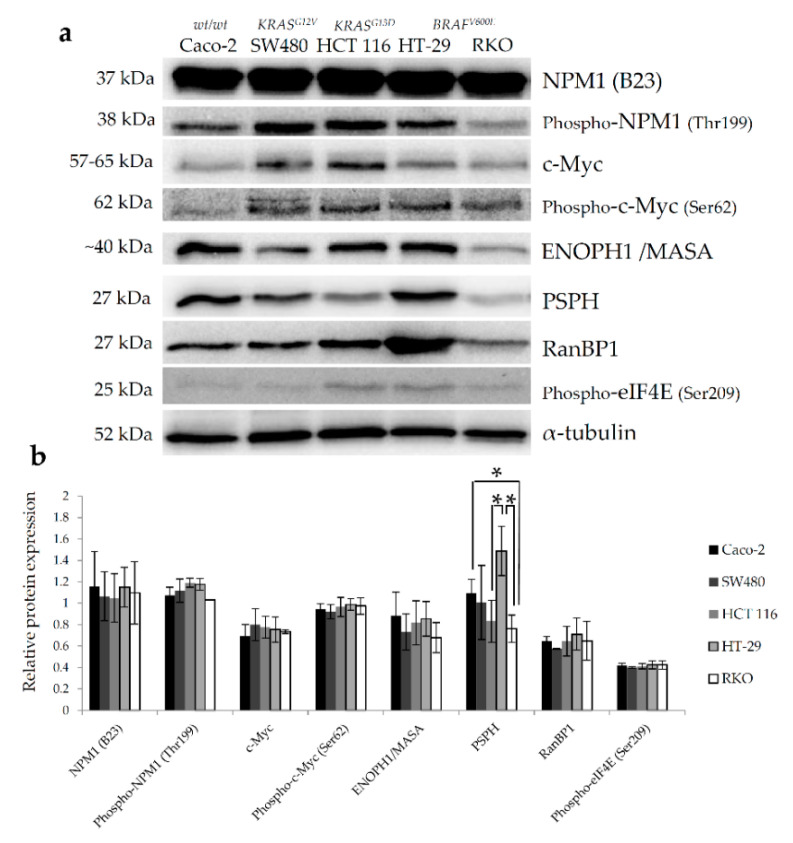
Western blot analysis of baseline levels of protein candidates chosen from proteomic dataset of up-regulated proteins in BRAFV600E mutant colon cancer cells following bioinformatics analyses. (**a**) Representative western blot images showing the baseline expression levels of selected proteins in two BRAFV600E mutant colon cancer cell lines harboring WT KRAS (HT-29 and RKO), two WT BRAF cell lines carrying KRAS mutations (HCT 116 and SW480) and a double WT colon cancer cell line (Caco-2). α-Tubulin was used as loading control. (**b**) Relative protein expression of selected proteins measured by densitometry analysis of individual bands using Quantity One software. Data represent mean and standard deviation obtained from three independent biological experiments. α-Tubulin was used as loading control. Statistical significance where *p* < 0.05 is denoted with an asterisk.

Baseline expression levels of selected protein targets were investigated by western blot in two BRAFV600E mutant colon cancer cell lines harboring WT KRAS (HT-29 and RKO), two WT BRAF cell lines carrying KRAS mutations (HCT 116 and SW480) and a double WT colon cancer cell line (Caco-2). Amongst all studied proteins, only the expression levels of NPM1 and phospho-c-Myc (Ser62) showed a trend towards an increase in both BRAF-mutated cell lines (Figure 3), albeit statistically non-significant when compared to other cell lines. Interestingly, a statistically significant difference was observed with up-regulated levels of PSPH in HT-29 cells in comparison with other cell lines carrying wild-type BRAF (Figure 3). However, this pattern of PSPH expression was not replicated in the RKO cell line, which could be, at least partially, explained by the difference in the mutational status of the *p53* gene between these two cell lines, which is critically involved in the regulation of amino acid metabolism in cancer cells [17].

**Figure 4:**
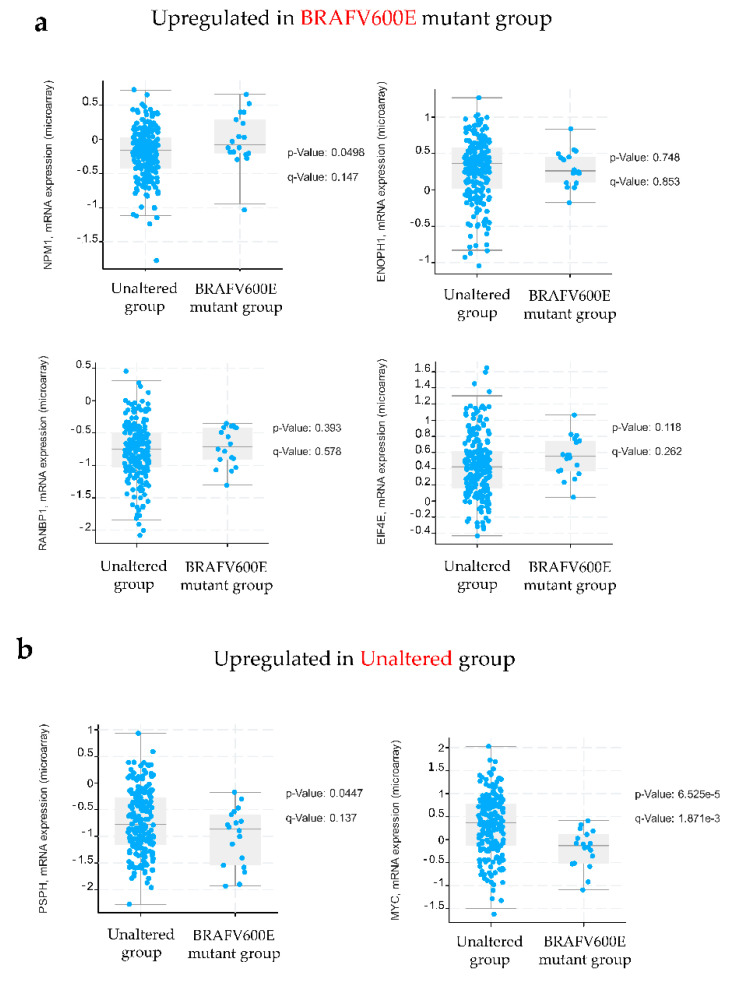
Validation of selected protein candidates in the Cancer Genome Atlas (TCGA) database. Shown here are mRNA expression levels corresponding to selected proteins using the colorectal adenocarcinoma dataset from the TCGA database in cBioPortal that included 20 cases with the BRAFV600E mutation and 200 cases without an indicated mutation (unaltered group). A *p*-value < 0.05 was considered statistically significant.

Next, we analyzed the mRNA expression profiles of selected targets in BRAF mutant colon cancer in The Cancer Genome Atlas (TCGA) colon adenocarcinoma cohort using cBioPortal and found that only the *NPM1* mRNA expression level was significantly increased (*p* = 0.0498) in BRAFV600E mutant colon cancer in comparison with WT BRAF (Figure 4). Although not significant, mRNA expression levels of *ENOPH1*, *RANBP1* and *EIF4E* were higher in BRAFV600E mutant colon cancer than in the unaltered group. An opposite trend in the mRNA expression was observed for *MYC* and *PSPH* whose gene expression was higher in the unaltered group (Figure 4). The discrepancy between the results obtained for *MYC* and *PSPH* at the protein level revealed by western blot and their respective mRNA expression levels retrieved from the TCGA database could be, at least partially, ascribed to different biological sources (cells vs. tissues) and to a poor correlation between mRNA and protein levels [18].

Altogether, data from in vitro and in silico studies indicate an increased abundance of nucleophosmin in BRAF mutant colon cancer. 

### 2.4. Nucleophosmin and c-Myc Mediate Resistance to BRAFV600E Inhibition by PLX4032 in BRAFV600E Mutant Colon Cancer Cell Lines

In order to investigate the role of nucleophosmin and its interaction partner c-Myc in the response and development of acquired resistance to BRAFV600E inhibition by vemurafenib (PLX4032), we measured the expression levels of NPM1, c-Myc and their phosphorylated forms p-NPM1 (Thr199) and p-c-Myc (Ser62) in sensitive colon cancer cell lines HT-29 and RKO and their vemurafenib-resistant counterparts HT-29r and RKOr, respectively, after exposure to cytotoxic concentrations (IC_50_) of PLX4032 (Supplementary Table S5) for 24, 48 and 72 h.

**Figure 5:**
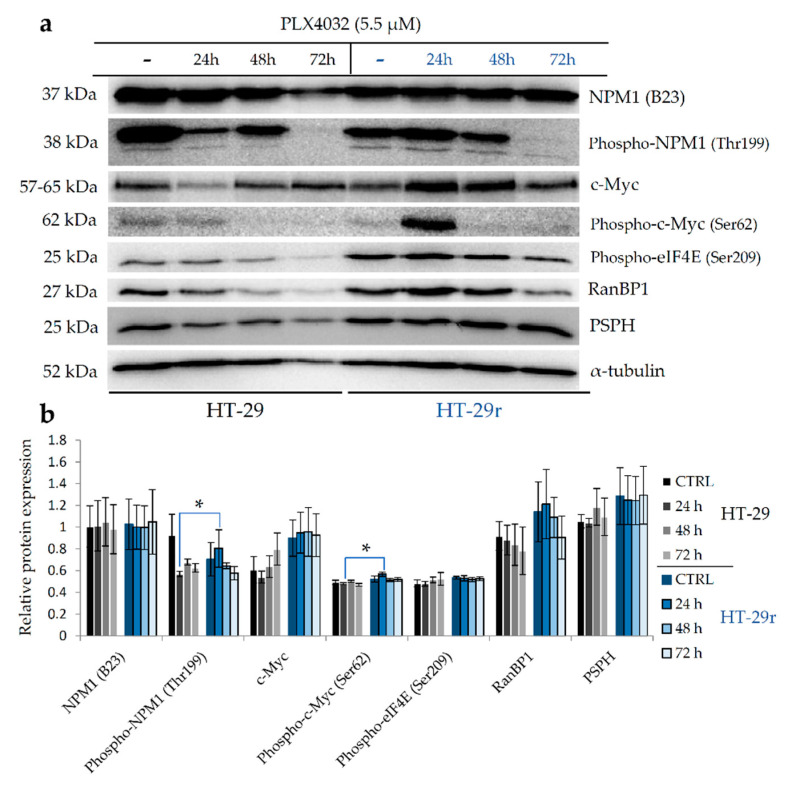

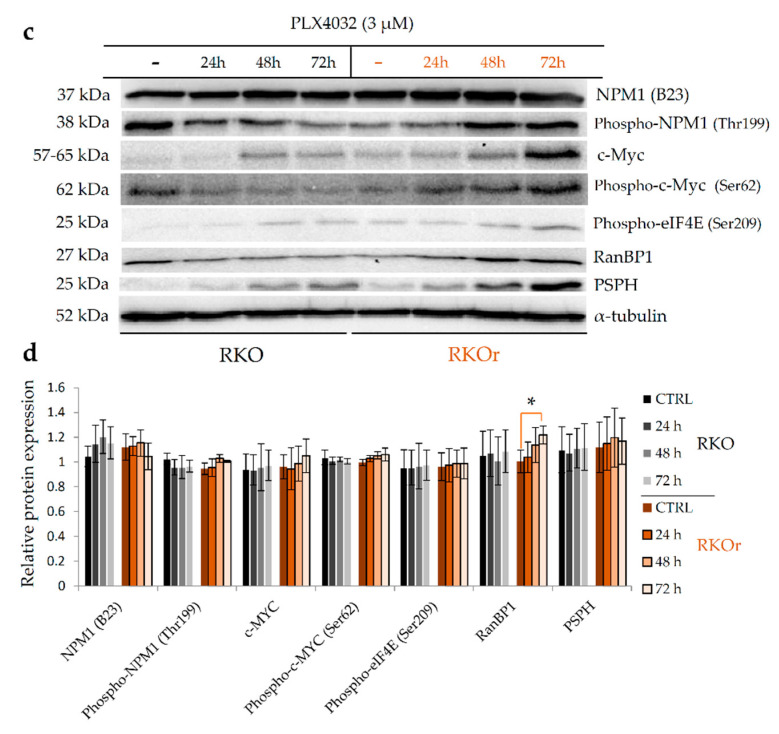
Western blot analysis of relative expression of selected proteins in BRAFV600E-mutated HT-29 (**a**,**b**) and RKO (**c**,**d**) sensitive and vemurafenib (PLX4032)-resistant colon cancer cell lines following the exposure to cytotoxic concentrations of PLX4032 at different time points. Densitometry analysis of western blot bands was carried out by Quantity One software to calculate relative abundance of selected proteins in parental and resistant HT-29 (**b**) and RKO (**d**) cells. Data represent mean and standard deviation obtained from two independent biological experiments with two technical replicates. α-Tubulin was used as loading control. Statistical significance where *p* < 0.05 is denoted with an asterisk.

Although PLX4032 did not have obvious effects on the expression profiles of NPM1 in both parental cell lines and their resistant counterparts, more immense alterations in the expression level of p-NPM1 (Thr199) were observed upon treatment with PLX4032. Specifically, parental HT-29 cells responded to PLX4032 by dramatically reducing the level of p-NPM1 (Figure 5a,b). On the contrary, PLX4032 induced a statistically significant increase in the expression level of p-NPM1 in resistant HT-29r cells after 24 h when compared to sensitive cells. A similar pattern of p-NPM1 expression in response to PLX4032 was detected in RKO cells (Figure 5c,d). Thus, in parental cells treated with PLX4032, there was a trend toward a consistent decrease in the expression level of p-NPM1, as opposed to resistant cells that gradually up-regulated their levels of p-NPM1, peaking at 48 h after the treatment. Altogether, treatment with PLX4032 resulted in a surge in p-NPM1 expression in both resistant cell lines, although with a different magnitude and temporal dynamics. As previously reported, phosphorylation of NPM1 by CDK2-cyclin E on threonine 199 triggers its dissociation from unduplicated centrosomes in the G1 phase, which promotes initiation of centrosome duplication [19]. With this in mind, it is tempting to believe that nucleophosmin might regulate the resistance to PLX4032 via the mechanisms that propel centrosome duplication. 

RanBP1 constitutes a component of the Ran–CRM1 complex that regulates centrosome duplication by controlling NPM1 cytoplasmic translocation and its association with centrosomes [20]. RanBP1 could inhibit the function of CRM1, resulting in NPM1 dissociation from the centrosomes and initiation of centrosome duplication. Treatment of parental HT-29 cells with PLX4032 gave rise to a sustained decline in the RanBP1 expression levels (Figure 5a,b). Importantly, the expression of RanBP1 was markedly increased in resistant HT-29r cells in comparison with parental cells peaking at 24 h after the treatment, which paralleled the expression pattern of p-NPM1 in resistant HT-29r cells exposed to PLX4032. Although parental RKO cells did not exert pronounced alterations in the RanBP1 expression profile within the tested timeframe, resistant RKOr cells did show a consistent increase in the RanBP1 expression levels with a statistical significance after 72 h of treatment (Figure 5c,d). The increased regulation of RanBP1 expression in resistant cell lines in response to PLX4032 challenge further supports the relevance of the mechanisms regulating initiation of centrosome duplication in the development of drug resistance in BRAF-mutated colon cancer cells.

The expression and activity of c-Myc, a common transcriptional regulator of NPM1 and RanBP1 and an interaction partner of NPM1, were also differentially regulated between sensitive and resistant cell lines after the PLX4032 challenge. The levels of c-Myc were generally higher in resistant HT-29r cells before and after the exposure to PLX4032 when compared with sensitive HT-29 cells (Figure 5a,b). Importantly, a statistically significant increase in the p-c-Myc level was observed in HT-29r cells after 24 h of treatment when compared to sensitive cells. Similarly, a trend towards a sustained increase in the levels of c-Myc and p-c-Myc peaking at 72 h after the exposure to PLX4032 was observed in resistant RKOr cells when compared to their sensitive counterpart (Figure 5c,d).

c-Myc plays a central role in the regulation of cancer cell metabolism including amino acid metabolism, where it functions as a positive regulator of the serine synthesis pathway. Thus, the expression of the enzyme catalyzing the last step in the biosynthesis of serine, namely, phosphoserine phosphatase (PSPH), is positively regulated by c-Myc at the gene and protein levels, and PSPH seems to be important for the oncogenic function of c-Myc in vitro and in vivo [16]. In line with this, the PSPH expression pattern measured in resistant cell lines exposed to PLX4032 paralleled the expression and activity of c-Myc (Figure 5). Thus, the expression levels of PSPH were generally higher in HT-29r before and after the PLX4032 challenge when compared to HT-29 cells, whereas RKOr cells exerted a trend towards increased levels of PSPH after the treatment with PLX4032 relative to sensitive RKO cells. 

Previous studies have shown altered levels of eIF4E in response to changes in the c-Myc levels and provided evidence to support the transcriptional activation of eIF4E by c-Myc [15]. We found that the levels of p-eIF4E (Ser209) did not change markedly in response to PLX4032 over the treatment period of 72 h in resistant and parental cell lines (Figure 5), and that its expression levels were not remarkably different between responsive and non-responsive cell lines. This finding led us to conclude that eIF4E is not critically involved in the regulation of the drug response and resistance to BRAF inhibition in BRAFV600E mutant colon cancer cells.

Collectively, our data put forward the possibility that molecular events associated with centrosome duplication, mitosis, transcription machinery and serine metabolism could play an important role in determining the responsiveness of BRAF mutant colon cancer cells to BRAF inhibition by PLX4032. Given the central regulatory roles of NPM1 and c-Myc in these processes, their targeting could provide novel opportunities to counteract drug resistance in BRAF mutant colon cancer.

### 2.5. Tumour Tissues from BRAF-Mutated Colon Adenocarcinoma Patients Have Significantly Increased Abundance of Cytoplasmic p-NPM1 (Thr199)

**Figure 6:**
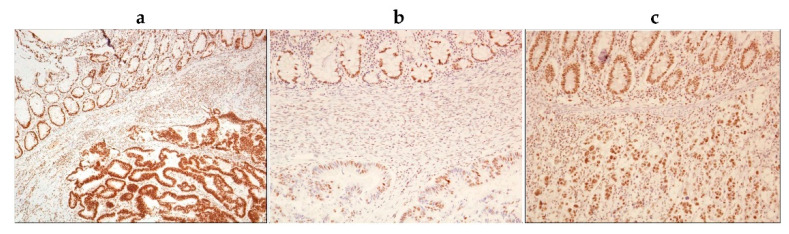
Representative microphotographs of immunohistochemical staining of phospho-nucleophosmin (Thr199) in colonic adenocarcinomas differing in BRAF mutational status. (**a**) Upper part of the picture showing normal colonic crypts, with moderate nuclear staining. Below is colonic adenocarcinoma with BRAF mutation showing strong nuclear and cytoplasmic staining; (**b**) upper part of the picture showing normal colonic crypts, with moderate nuclear staining. Below is colonic adenocarcinoma with KRAS mutation showing weak nuclear and negative cytoplasmic staining; (**c**) upper part of the picture showing normal colonic crypts, with moderate nuclear staining. Below is colonic adenocarcinoma BRAF and KRAS wild types showing moderate nuclear and negative cytoplasmic staining. Magnification 100× (6**a**) and 200× (6**b** and 6**c**).

Since our data indicate the role of p-NPM1 (Thr199) in regulating the resistance of BRAF-mutated colon cancer cells to BRAF inhibition, we hypothesized that tumor tissues from BRAF-mutated colon cancer patients, who present with an aggressive clinical phenotype and have a generally poor response to systemic chemotherapy, could have an increased abundance of p-NPM1. In order to verify this presumption, we examined the levels of *p*-NPM1 (Thr199) in tumor tissues from colon adenocarcinoma (CA) patients with different BRAF mutational statuses using immunohistochemistry. We observed that all CAs had positive nuclear staining of nucleophosmin (phospho threonine 199), but with different staining intensities, and none of the tissue samples were negative (Figure 6, Table 4, Supplementary Table S6). When we analyzed the cytoplasm of tumor cells, we observed that there was a greater difference in staining intensity, from negative to strong.

In the group of BRAF-mutated CAs, the nuclear staining intensity of nucleophosmin (phospho T199) was predominantly strong. In addition, BRAF-mutated CAs also had a strong cytoplasmic staining intensity. In colonic adenocarcinomas with the KRAS mutation, nuclei were moderately stained with a weak to negatively stained cytoplasm. Colonic adenocarcinomas that were BRAF and KRAS wild types had moderate to strong nuclear staining, and weak to negative cytoplasmic staining (Figure 6, Table 4, Supplementary Table S6).

When we compared nuclear staining of nucleophosmin (*p*-T199) in BRAF-mutated vs. BRAF wild-type CAs (with and without the KRAS mutation), there was a statistically significant difference between these two groups (Student’s *t*-test, *p* = 0.009), even in such a small sample size (Table 4). Importantly, a greater difference was seen in the cytoplasmic staining intensity of nucleophosmin (p-T199). BRAF-mutated CAs mostly had a strong staining intensity of their cytoplasm compared to BRAF wild-type CAs, which stained mostly weak to negative. This observation was also statistically significant (Student’s *t*-test, *p* = 0.005). Altogether, these results point to a significantly increased abundance of cytoplasmic *p*-NPM1 (Thr199) in tumor tissues from BRAF-mutated in comparison with wild-type BRAF CA patients. 

### 2.6. Pharmacological Inhibition of Oncogenic Nucleophosmin/MYC Axis Restores Sensitivity of BRAF Mutant Colon Cancer Cells to PLX4032

Prompted by the findings that resistant BRAF-mutated colon cancer cells exposed to cytotoxic doses of PLX4032 express an increased activity of nucleophosmin associated with the promotion of centrosome duplication concomitant with an increased stability and activity of its binding partner, c-Myc, we sought to further investigate whether pharmacological targeting of NPM1 and c-Myc could increase the efficacy of PLX4032 in resistant cell lines. NPM1 was specifically inhibited by NSC348884, a small molecule inhibitor that targets nucleophosmin oligomer formation, leading to consequent impairment of its function [21]. Resistant cell lines RKOr and HT-29r were pre-treated with either IC_50_ or 2 × IC_50_ concentrations of NSC348884 for 2 and 4 h followed by culturing the cells in the presence of five different concentrations of PLX4032 (10^−8^ to 10^−4^ µM) for a total period of 72 h. In both resistant cell lines, pre-treatment with IC_50_ concentrations of NSC348884 for 2 h induced an obvious reduction in cell viability in comparison with single-agent PLX4032, and this effect was markedly potentiated in both resistant cell lines after pre-treatment with 2 × IC_50_ concentrations of NSC348884, which strongly suppressed cell proliferation, as evidenced by more than a two-fold decrease in IC_50_ values in comparison with single-agent PLX4032 (Table 5). Importantly, the longer pre-treatment period of 4 h in the presence of 2 × IC_50_ concentrations of NSC348884 produced a dramatic reduction in cell viability in both resistant cell lines in comparison with a single treatment with PLX4032.

We next examined the effects of pre-treatment with IZCZ-3 on the response of resistant cell lines to PLX4032. IZCZ-3 stabilizes the G-quadruplex structure in the c-Myc promoter, resulting in the suppression of c-Myc transcription [22]. Generally, pre-treatment with IZCZ-3 produced a more potent anti-proliferative effect in combination with PLX4032 in resistant RKO cells than in resistant HT-29 cells (Table 6), which could be, at least partially, ascribed to the fact that, unlike HT-29 cells, RKO cells harbor the wild-type *p53* gene which represses c-Myc transcription and suppresses c-Myc-induced cell proliferation [23]. Thus, the inhibitory effect of IZCZ-3 on c-Myc in RKO resistant cell line could be additionally augmented by a negative feedback regulation of c-Myc expression by WT *p53*, resulting in a marked cytostatic effect, particularly after pre-treatment with 2 × IC_50_ concentration of IZCZ-3 (Table 6).

As for HT-29 resistant cells that carry the mutant *p53* gene, more pronounced anti-proliferative effects in combination with PLX4032 were observed only with 2 × IC_50_ concentration of IZCZ-3 after an 8-h pre-treatment (Table 6). The modest chemosensitization effect of IZCZ-3 in HT-29r cells could be potentially explained by the previous findings revealing that mutant *p53* induces the expression of the endogenous *c-myc* gene via the activation of the *c-myc* promoter [24]. With this in mind, we cannot rule out the possibility that mutant *p53*-dependent induction of c-Myc in HT-29 resistant cells overrides its inhibition by IZCZ-3 to enable cellular proliferation and survival. Targeting c-Myc with IZCZ-3 may not thus be sufficient to maximize the cytotoxic effects of vemurafenib in resistant cells due to the possible impact of the mutational status of the *p53* gene, which does not seem to be the case with targeting NPM1 by NSC348884. Importantly, the magnitude of the cytostatic effects induced by NSC348884, especially after a longer treatment period with 2 × IC_50_ concentration, was not reached with IZCZ-3. These findings hint that nucleophosmin might be a putative target to overcome resistance to vemurafenib in BRAF-mutated colon cancer cells regardless of the presence of other oncogenic mutations.

**Figure 7:**
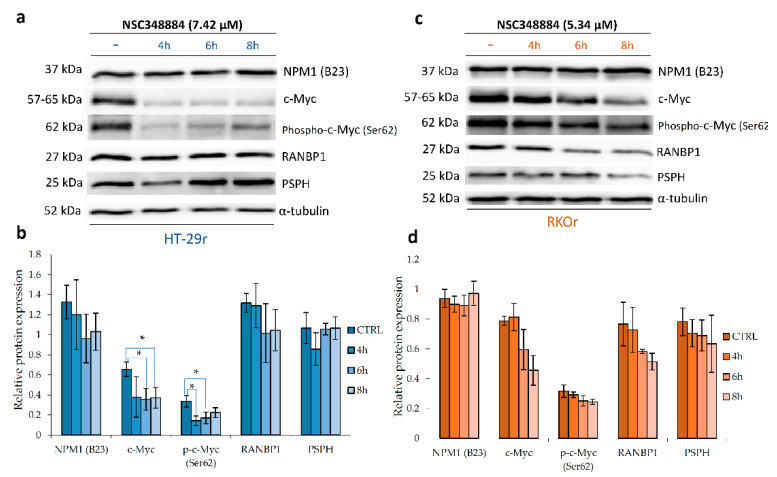
Western blot analysis of relative protein expression of nucleophosmin, c-Myc and its transcriptional targets RanBP1 and phosphoserine phosphatase (PSPH) in vemurafenib-resistant HT-29r (**a**,**b**) and RKOr (**c**,**d**) colon cancer cells with BRAFV600E mutation after the treatment with cytotoxic concentrations of nucleophosmin inhibitor NSC348884 for indicated time periods. Densitometry analysis of western blot bands was carried out by Quantity One software to calculate relative protein abundance (**b**,**d**). Data represent mean and standard deviation obtained from two independent biological experiments with two technical replicates. α-Tubulin was used as loading control. Statistical significance where *p* < 0.05 is denoted with an asterisk.

In order to further investigate whether the observed anti-proliferative effects induced by pharmacological inhibition of NPM1 could be attributed to suppression of its binding partner, c-Myc, and abrogation of c-Myc-driven cellular events, we carried out western blot analysis of resistant cell lines treated with 2 × IC_50_ of NSC348884 for 4, 6 and 8 h. We found that inhibition of NPM1 oligomerization by NSC348884 significantly suppressed the expression of c-Myc and p-c-Myc in HT-29r cells (Figure 7). In addition, a trend towards a reduction in c-Myc and p-c-Myc levels in a time-dependent manner was also observed in RKOr cells after the treatment with NSC348884. The observed decline in c-Myc and p-c-Myc levels was accompanied by an obvious decrease in RanBP1 levels in both resistant cell lines as early as 6 h after the treatment with NSC348884. Similarly, PSPH levels descended in both resistant cell lines exposed to NSC348884, although with different temporal dynamics and intensities (Figure 7).

To sum up, findings from this study indicate that inhibition of NPM1 oligomerization increases the responsiveness of resistant cells to vemurafenib by mechanisms that might include down-regulation of c-Myc transcription and activity, leading to attenuation of downstream signaling regulated by RanBP1 and PSPH.

## 3. Discussion

In this paper, we described up-regulated proteomic features that could be associated with the BRAFV600E mutation in colon cancer cells. Previous studies mainly focused on identifying characteristic gene expression patterns of BRAFV600E-mutated colon cancer by examining differences between oncogenic BRAF vs. either the KRAS mutation [4,7,25] or double wild-type BRAF/KRAS [5,6]. However, our study goes beyond previous reports by contrasting the BRAF mutant with both BRAF wild-type/KRAS mutant and double wild-type BRAF/KRAS phenotypes to circumvent the possibility of identifying overlapping features between oncogenic BRAF- and KRAS-driven biology. The results from our bioinformatics analyses of the up-regulated proteomic dataset partially replicate previous findings from gene expression analyses in BRAFV600E mutant colon cancer by revealing some common molecular events. Specifically, actin cytoskeleton organization was identified as an important process associated with up-regulated proteins and genes in our and similar studies, respectively, which suggests that activating the BRAFV600E mutation affects the cytoskeletal structure and dynamics in colon cancer [7,25]. A similar conclusion was reached in melanoma cells by showing that BRAFV600E mutant expression contributes to oncogene-mediated reorganization of the actin cytoskeleton and focal adhesions [26]. Importantly, remodeling of the actin cytoskeleton was demonstrated in BRAFV600E mutant melanoma cells with acquired resistance to BRAF inhibitor vemurafenib, and depletion of TESK1, a kinase that regulates actin cytoskeleton dynamics and reversed resistance to vemurafenib [27]. With this in mind, inhibition of actin remodeling could also prove to be a promising strategy to overcome resistance to vemurafenib in BRAF mutant colon cancer.

Another interesting result emerging from our study relates to increased baseline levels of phosphoserine phosphatase (PSPH) in HT-29 cells, an enzyme regulating serine biosynthesis. This finding is in good agreement with a previous study showing the up-regulation of the key proteins central to the serine biosynthesis pathway, including PSPH, in BRAFV600E mutant relative to wild-type BRAF colon cancer cells [28]. In addition, the BRAFV600E mutation status was shown to be associated with the serine biosynthesis pathway in other cancer types including papillary thyroid carcinoma, where higher expression of PSPH and other serine metabolism-related proteins was found in BRAFV600E-mutated tumors [29]. Importantly, we demonstrated that PSPH expression levels rose following the exposure to vemurafenib in resistant cell lines derived from HT-29 and RKO in a time-dependent manner, and this effect was not observed in parental cells. This finding further supports the role of serine biosynthesis in the development of resistance to vemurafenib in BRAF-mutated colon cancer cells. Similar to our findings, the role of the serine biosynthesis pathway in the mechanisms underlying the resistance to BRAFV600E inhibitors has been previously reported in melanoma, pancreatic and non-small cell lung cancer cells [30]. 

Besides some common and previously described features of BRAFV600E mutant colon cancer, our study reveals, for the first time, increased regulation of nucleophosmin (NPM1) expression in BRAFV600E-mutated colon cancer. NPM1 is involved in diverse cellular processes including ribosome biogenesis, centrosome duplication, protein chaperoning, histone assembly, cell proliferation and regulation of tumor suppressors p53/TP53 and ARF. High expression of NPM1 was previously shown to correlate with lymph node metastasis in colon cancer patients and to promote in vitro colon cancer cell proliferation, migration and invasion [31]. However, its involvement in the BRAFV600E-regulated cellular landscape in cancer has not been previously reported. We observed a trend towards an increased basal expression of the NPM1 protein in BRAF-mutated colon cancer cell lines in comparison with the cells carrying either WT BRAF/mutant KRAS or double WT BRAF/KRAS. Furthermore, analysis of the mRNA expression profile data from colon adenocarcinoma patients in the TCGA database revealed significant up-regulation of the *NPM1* gene in BRAFV600E-mutated colon cancer. Importantly, we detected significantly stronger nuclear and, in particular, cytoplasmic staining of phospho-NPM1 (Thr199) in tumor tissue specimens from BRAFV600E-mutated in comparison with KRAS mutant/BRAF wild-type and double wild-type KRAS/BRAF colonic adenocarcinomas using immunohistochemistry. Phosphorylation of NPM1 on Thr199 by cyclin-dependent kinase 2 (CDK2)/cyclin E is critical for the physical separation of the paired centrioles, which triggers the initiation of centrosome duplication [19]. The role of NPM1 in regulating mitosis was corroborated in different studies showing that cancer cells undergoing mitosis have a high level of NPM1 phosphorylated on Thr199 predominantly localized to the cytoplasm and the centrosome of dividing cells [32,33]. Increased expression of NPM1 phosphorylated on Thr199 in tumor tissues from BRAF mutant colon cancer patients could be thus related to anomalies in the centrosome cycle leading to centrosome amplification, which is a common event in colon cancer linked with mutations in several cancer-associated genes including BRAF [34]. Observations from our study concur with data from previous reports that pointed to aberrant centrosomal activity in BRAFV600E-mutated colon cancer by revealing an increased expression of the genes encoding for several different centrosomal proteins in BRAF mutant colon cancer cells and tumor tissues [25].

The finding of increased expression and activity of NPM1 in BRAF-mutated colon cancer prompted us to examine its potential role in modulating the response and resistance to BRAFV600E inhibitor vemurafenib. While BRAFV600E-mutated parental cell lines reduced their levels of p-NPM1 (Thr199) following vemurafenib challenge, their resistant counterparts responded to vemurafenib exposure by ascending the levels of p-NPM1 (Thr199), which points to the promotion of the centrosome duplication cycle and mitosis as potential mechanisms underlying vemurafenib resistance. The same pattern of RanBP1 expression recorded in resistant cell lines after the treatment with vemurafenib lends further support to the idea that the development of resistance to vemurafenib in BRAF-mutated colon cancer cells is associated with centrosome amplification and mitotic progression. RanBP1, a RAN partner with the highest levels in G2 and mitosis, regulates mitotic spindle assembly by controlling the spatial distribution and the extent of mitotic Ran-GTP production and provides the spatial control of specific factors regulating mitotic microtubule function [35,36]. Similar to our findings, a previous study also identified mitosis as a potential vulnerability of BRAFV600E-mutated colon cancer cell lines by revealing *RANBP2* as the gene indispensable for survival of BRAFV600E colon cancer cell lines with no effects on the viability of wild-type KRAS/BRAF colon cancer cells [6]. Depletion of *RANBP2* in BRAFV600E mutant colon cancer cells impaired spindle formation and prolonged mitosis, ultimately inducing cell death, which led the authors to hypothesize that BRAF mutant colon cancer cells could be sensitive to microtubule-disrupting agents such as vinorelbine, and this presumption was indeed confirmed in vitro and in vivo [6].

Previous studies adduced a wealth of evidence to support the correlation between the expression of NPM1 and c-Myc [13]. NPM1 binds the *c-myc* gene promoter, resulting in transcriptional regulation of the *c-myc* gene [37]. In addition, the endogenous and exogenous NPM1 protein has the ability to directly interact with the c-Myc protein to regulate the expression of c-Myc target genes at the promoter [38]. NPM1 was shown to induce c-Myc transcriptional activity resulting in c-Myc-induced hyperproliferation and transformation [38]. The clue that NPM1 regulates c-Myc localization and function arose from a study showing that NPM1 facilitates the localization of the c-Myc protein to nucleoli and enhances its ability to induce rRNA synthesis involved in ribosome biogenesis [39]. Here, we have shown that an increased basal expression of NPM1 in BRAF mutant colon cancer cell lines coincides with an increased expression level of phospho-c-Myc (Ser62). Importantly, we detected an upsurge in the expression levels and activity of c-Myc in both resistant cell lines after the PLX4032 challenge, which implies that c-Myc plays an important role in modulating vemurafenib resistance in BRAF mutant colon cancer cells. Similarly, a study in BRAF mutant melanoma indicated that c-Myc is reactivated at the gene and protein levels in tumor tissues and cell lines with acquired resistance to BRAF inhibitors [40]. The same authors reported that resistant cells have higher MYC levels in comparison with their parental counterparts. Expectedly, MYC knockdown increased the sensitivity of resistant cells to BRAF inhibition. Our results are partially in accordance with these findings by revealing that inhibition of c-Myc transcription by IZCZ-3 selectively induced a marked increase in the response to vemurafenib in BRAFV600E mutant colon cancer cells carrying wild-type p53, which suggests that the chemosensitizing effect of c-Myc inhibition might be governed by the functional status of the *p53* gene. Inhibition of the nucleophosmin function by NSC348884 in vemurafenib-resistant colon cancer cell lines with a BRAF mutation potentiated the cytotoxic effects of vemurafenib in a concentration- and time-dependent manner, regardless of the *p53* status, to an extent that was not observed with c-Myc inhibition. These results posit that NPM1 might be a putative target to restore sensitivity to BRAF inhibitors in colon cancer. Furthermore, inhibition of NPM1 gave rise to attenuation of c-Myc expression and activity in resistant cells, leading to suppression of its transcriptional targets RanBP1 and PSPH that regulate the centrosomal cycle and serine biosynthesis, respectively, which confirms previous literature data on the NPM1-regulated expression and activity of c-Myc, and puts forward the idea that inhibition of multiple targets resulting from blockade of NPM1 activity could be an efficient approach to increase the efficacy of vemurafenib in resistant BRAF mutant colon cancer cells, at least under in vitro conditions.

In conclusion, we found that NPM1 expression and activity potentially associated with the regulation of centrosome duplication and mitotic progression are specifically increased in BRAFV600E mutant colon cancer cells and tumor tissues from BRAF-mutated colon adenocarcinoma patients. Cellular processes linked with the initiation of centrosome duplication, mitosis and serine metabolism could be possibly involved in determining the responsiveness of BRAF mutant colon cancer cells to BRAF inhibition by vemurafenib, where nucleophosmin and its interaction partner and downstream target c-Myc play central regulatory roles. Furthermore, our data argue that pharmacological inhibition of the NPM1 function could restore the sensitivity of vemurafenib-resistant colon cancer cells with the BRAFV600E mutation by mechanisms that include down-regulation of c-Myc expression and activity and consequent suppression of its transcriptional targets RanBP1 and phosphoserine phosphatase regulating centrosome duplication and serine biosynthesis, respectively. Findings from this study point to the therapeutic potential of targeting the NPM1/c-Myc axis in BRAF-mutated colon cancer and provide a framework to devise novel strategies for counteracting the resistance to BRAF inhibition in colon cancer.

## 4. Materials and Methods

### 4.1. Cell Culturing and Development of Vemurafenib-Resistant Colon Cancer Cell Lines

Human colon carcinoma cell lines Caco-2 (BRAF^wt^/KRAS^wt^), SW480 (BRAF^wt^/KRAS^G12V^), HCT 116 (BRAF^wt^/KRAS^G13D^) and HT-29 and RKO (BRAF^V600E^/KRAS^wt^) were purchased from the ATCC and maintained in Dulbecco′s Modified Eagle′s Medium (DMEM) or Minimum Essential Medium (MEM) supplemented with 10% fetal bovine serum (FBS), 2 mM L-glutamine, penicillin (100 U/mL) and streptomycin (100 µg/mL) (Capricorn Scientific, Ebsdorfergrund, Germany) in a humified atmosphere with 5% CO_2_ at 37 °C.

In order to eliminate molecular features of resistance that might be cell line-specific, we developed two vemurafenib (PLX4032)-resistant colon cancer cell lines derived from HT-29 and RKO cell lines by exposing the cells to successively increasing concentrations of PLX4032 (MedChemExpress, Monmouth Junction, NJ, USA) in a period of about 6 months until a clinically relevant dose (11.52 µM) [41] was reached. Established resistance phenotypes were confirmed by the MTT assay showing an increase in the IC_50_ values by 8- and 10-fold in the resistant HT-29 and RKO cells, respectively, in comparison with their sensitive counterparts (Supplementary Table S5).

### 4.2. Cell Viability Assay

Cell viability was assessed using the MTT assay. Briefly, cells were seeded onto 96-well microtiter plates at a seeding density of 3000 cells/well. The following day, cells were treated with test agents in five 10-fold serial dilutions (10^–4^–10^–8^ µM) and further incubated for 72 h. MTT assay was performed according to the manufacturer’s instructions (Sigma-Aldrich, St. Louis, MO, USA). After the completion of the treatment period, cells were incubated with MTT reagent for 3 h in the dark followed by the addition of dimethyl sulfoxide (DMSO, Sigma-Aldrich, St. Louis, MO, USA). Absorbance was measured at 570 nm using a Sunrise Absorbance microplate reader (Tecan Life Sciences, Männedorf, Switzerland). Inhibitory and lethal concentrations (IC_50_ and LC_50_, respectively) were calculated using linear regression analysis.

Pre-treatment of cells with either NSC348884 (MedChemExpress, Monmouth Junction, NJ, USA) or IZCZ-3 (MedChemExpress, Monmouth Junction, NJ, USA) was carried out at previously determined IC_50_ and 2x IC_50_ values of each agent for indicated time periods. Following the treatment, cells were washed twice with fresh medium and further treated with five 10-fold serial dilutions of PLX4032 for 72 h, and cell viability was assessed using the MTT assay as described above.

### 4.3. Two-Dimensional Gel Electrophoresis and Image Analysis

Cells were lysed in 2-DE lysis buffer containing 7M urea, 2M thiourea, 4% CHAPS and 1% DTT (Sigma-Aldrich, St. Louis, MO, USA USA) supplemented with protease inhibitor cocktail (Roche, Switzerland). A total of 800 µg of proteins was solubilized in 2-DE rehydration buffer (7M urea, 2M thiourea, 4% CHAPS, 1% DTT and 0.2% Bio-Lyte ampholyte (Bio-Rad, Hercules, CA, USA), loaded onto 17 cm pH 4–7 IPG strips and subjected to isoelectric focusing on PROTEAN IEF cell (Bio-Rad). The IEF conditions were as follows: 50 V for 12 h, 200 V for 15 min, 500 V for 1 h, 500–10,000 for 3 h and 10,000 V for 5 h. In the second dimension, proteins were resolved by 12% SDS-polyacrylamide gels by PROTEAN II XL cell (Bio-Rad, Hercules, CA, USA). Gels were stained with Coomassie Blue G-250 (Sigma-Aldrich, St. Louis, MO, USA) overnight, and after washing in miliQ water, gel images were taken by ChemiDoc XRS+ Imager (Bio-Rad, Hercules, CA, USA). The 2-DE gel image analysis was carried out using Progenesis SameSpots 4.0 software (TotalLab, Newcastle upon Tyne, United Kingdom). The experiment was performed in four individual biological replicates for each cell line. ANOVA followed by post hoc Tukey’s test was carried out to identify statistically significant differences in protein abundance between the datasets obtained for the three cell lines differing in their BRAF mutational status.

### 4.4. MALDI-TOF/TOF Mass Spectrometry Analysis

Each sample was mixed with matrix solution containing α-cyano-4-hydroxycinnamic acid (0.3 g/L CHCA in a solution containing 2:1 ethanol/acetone, *v*/*v*) at the ratio of 1:10. A total amount of 1 µL of the mixture containing sample/matrix solution was spotted onto the MALDI plate (AnchorChip 800 μm, Bruker Daltonics, Bremen, Germany) and kept at room temperature to allow crystallization to occur. An UltrafleXtreme MALDI-TOF/TOF mass spectrometer (Bruker Daltonics, Billerica, MA, USA) was used to perform MS analyses in the reflector mode in the m/z range of 700–3500 Da. The MS spectra were externally calibrated with the mixture of Peptide Calibration Standard and Protein Calibration Standard I (Bruker Daltonics, Billerica, MA, USA) at the ratio of 1:5. FlexControl 3.4 software (Bruker Daltonics, Billerica, MA, USA) was applied to acquire and process spectra. FlexAnalysis 3.4 (Bruker Daltonics, Billerica, MA, USA) was applied to perform protein database searches. Proteins were identified using the Mascot 2.4.1 search engine (Matrix Science, London, UK). The following search parameters were applied: enzyme: trypsin; fixed modifications: carbamidomethylation on cysteine; variable modifications: oxidation on methionine; protein mass: unrestricted; peptide mass tolerance: ±50 ppm; maximum missed cleavage: 2.

### 4.5. Bioinformatic Analyses

GO enrichment analysis using the DAVID functional annotation tool (https://david.ncifcrf.gov/, accessed on 20 May 2020) was used to elucidate the biological functions of proteins, where enriched GO terms with *p* < 0.05 were considered as statistically significant. The Reactome pathway database (https://reactome.org/, accessed 7 April 2021) was applied for pathway enrichment analysis.

Enrichment to identify the interactor proteins of the selected proteomic dataset prior to generation of the protein–protein interaction network was performed by DAVID using the following criteria: the threshold for enrichment was set to log fold change (FC) > 1.5 and *p* < 0.05.

The Search Tool for Retrieval of Interacting Genes (STRING) (http://string-db.org/, accessed on 27 May 2020) online tool was applied to construct the PPI network, where the confidence score was set to 0.900 (highest confidence). The PPI network was visualized by Cytoscape (https://cytoscape.org/, accessed on 27 May 2020), an open source software platform for visualizing complex networks. Each node corresponds to a protein, whereas the edges represent the interactions between proteins that contribute to the same biochemical function or pathway.

In order to analyze and select significant modules of the PPI network, the Molecular Complex Detection (MCODE) plugin of Cytoscape was employed with the following module identification criteria: node score cutoff = 0.2; degree cutoff = 2; maximum depth = 100; and k-core = 2 [42]. To further identify the hub proteins in selected significant modules (clusters), we used Cytohubba, a Cytoscape plugin.

### 4.6. Human Tissue Samples

Tissue samples of 21 colonic adenocarcinomas (CAs) (excluding rectal) were obtained from the archive of the Department of Pathology and Cytology, Clinical Hospital Center Rijeka, from 2015 to 2020. The diagnostic criteria of colonic adenocarcinoma were based on the WHO classification. All 21 CAs were tested for BRAF and KRAS mutations. Of 21 CAs, a total of 7 had the BRAFV600E mutation.

### 4.7. Immunohistochemistry Analysis

Immunohistochemical staining was performed on formalin-fixed, paraffin-embedded 4 µm tissue sections of colonic adenocarcinoma and stained with antibody against phosho-nucleophosmin (Thr199) antibody (Abcam). Tissue sections of 4 µm-thick paraffin blocks were placed on silane-coated slides and dried overnight at 37 °C. This was followed by deparaffinization with xylene three times over 10 min and then rehydration with 100% absolute ethyl alcohol for 5 min, 96% alcohol for 5 min and then 70% alcohol for 5 min. The rehydration procedure was followed by rinsing in distilled water for 10 min. Heat-induced pre-treatment was used to detect antigenic epitopes. Tissue slides for nucleophosmin detection were immersed in target retrieval solution (3 in 1) in a PT link apparatus for 20 min at a temperature of 97 °C. After cooling for 20 min at room temperature and rinsing in distilled water, the slides were ready for immunohistochemical staining. The “EnVision” immunohistochemical method with the REAL ENVISION DETECTION system on the Dako Cytomation, Autostainer plus, Glostrup, Denmark automated dye was used to determine protein expression according to the manufacturer’s instructions. The dilution of the primary antibody was 1:1000, and the incubation of the primary antibody was 30 min. The negative control in each tissue specimen consisted of the substitution of primary mAb with wash solution. We investigated nucleophosmin (NPM1) expression by immunohistochemistry in CA tissue sections divided into two groups: BRAFV600E mutant CAs (N = 7) and BRAF wild-type CAs (N = 14), which included KRAS wild-type (N = 6) or KRAS-mutated CAs (N = 8). For the tissue evaluation of NPM expression, each slide was semiqualitatively scored—estimation was based on the nuclear staining intensity (0—negative, 1—weak, 2—strong) and cytoplasmic staining intensity (0—negative, 1—weak, 2—strong), and the mean value was calculated.

### 4.8. Western Blot Analysis

Cells were seeded in 6-well plates at a density of 1.5 × 10^5^ cells per well and cultured for the indicated time period in the presence or absence of test agent. Cells were then lysed using RIPA buffer (25 mM Tris-HCl (pH 7.4), 1% NP-40, 0.5% Sodium Deoxycholate, 0.1% SDS, 150 mM NaCl) supplemented with protease and phosphatase inhibitor cocktails (Roche). A total of 50 µg proteins was resolved on 10% or 12% SDS polyacrylamide gels and transferred onto PVDF membranes (Bio-Rad, Hercules, CA, USA). Membranes were blocked in either 5% bovine serum albumin (BSA) or non-fat milk prepared in TBST and probed with primary antibodies against NPM1 (1:4000) from Sigma-Aldrich and p-NPM1 (Thr199), c-Myc, p-c-Myc (Ser62), ENOPH1, PSPH, RanBP1, p-eIF4E (Ser209), p-ERK1/2 (Thr202/Tyr204), p-MEK1/2 (Ser217/221), p-AKT (Ser473), p-cRAF (Ser338) and α-tubulin (1:1000) from Cell Signalling Technologies, overnight at 4 °C. The next day, membranes were washed with TBST and probed with secondary antibody goat anti-mouse or goat anti-rabbit (Cell Signalling Technologies, 1:2000). Protein bands were visualized using Amersham™ ECL™ Prime Western blotting Detection Reagent and Imagequant LASS 500 (GE Healthcare). Relative protein expression was analyzed by Quantity One 1-D Analysis Software (Bio-Rad, Hercules, CA, USA). Statistical analysis was performed by the two-tailed *t*-test, where a *p*-value of < 0.05 was considered statistically significant.

## Figures and Tables

**Table 1:** List of differentially up-regulated proteins with statistical significance (*p* < 0.05) in BRAFV600E mutant HT-29 cells in comparison to Caco-2 and SW480 colon cancer cells.

Spot Number	Accession ID	Protein Name *p*-Value (HT-29 vs. Caco-2; HT-29 vs. SW480)	Molecular Weight (kDa)	Peptide Matches	Sequence Coverage (%)	SCORE
**160**	MIC60_HUMAN	MICOS complex subunit MIC60 (0.002; 0.044)	83.60	4	10.00	307.41
**517**	K1C20_HUMAN	Keratin, type I cytoskeletal 20 (0.001; 0.001)	48.50	4	9.90	145.21
**678**	ROAA_HUMAN	Heterogeneous nuclear ribonucleoprotein A/B (0.010; 0.011)	36.20	2	3.90	60.25
**731**	NPM_HUMAN	Nucleophosmin (0.028; 0.016)	32.60	4	16.30	191.18
**717**	K2C1_HUMAN	Keratin, type II cytoskeletal 1 (0.009; 0.003)	66.00	1	2.50	44.11
**925**	TPM1_HUMAN	Tropomyosin alpha-1 chain (0.030; 0.001)	32.70	2	8.80	79.33
**983**	RANG_HUMAN	Ran-specific GTPase-activating protein (0.001; 0.0003)	23.47	16	47.00	76.00
**996**	UCHL3_HUMAN	Ubiquitin carboxyl-terminal hydrolase isozyme L3 (0.039; 0.023)	26.34	18	70.00	178.00
**1191**	ARI5B_HUMAN	AT-rich interactive domain-containing protein 5B (0.006; 0.002)	133.43	14	17.00	37.00
**985**	MIXTURE 1 SERB_HUMAN IF4E_HUMAN	Phosphoserine phosphatase Eukaryotic translation initiation factor 4E (0.040; 0.030)	25.16 25.31	16 14	62.00 48.00	100.00 85.00
**961**	YWHAZ _HUMAN	14-3-3 protein theta (0.043;0.061)	28.032	13	40.00	53.00
**1162**	NMES1_HUMAN	Normal mucosa of oesophagus-specific gene 1 protein (0.005; 0.004)	9.611	4	55.00	37.00
**855**	ENOPH_HUMAN	Enolase-phosphatase E1 (0.027; 0.0978)	28.90	6	34.10	378.18
**971**	YWHAQ_HUMAN	14-3-3 protein zeta/delta (0.073; 0.043)	27.899	21	38.00	114
**655**	CAPG_HUMAN	Macrophage-capping protein (0.0003; 0.077)	38.50	4	10.30	223.83

**Table 2:** List of differentially down-regulated proteins with statistical significance (*p* < 0.05) in BRAFV600E mutant HT-29 cells in comparison to Caco-2 and SW480 colon cancer cells.

Spot Number	Accession ID	Protein Name *p*-Value (HT-29 vs. Caco-2; HT-29 vs. SW480)	Molecular Weight (kDa)	Peptide Matches	Sequence Coverage (%)	SCORE
**183**	MOES_HUMAN	Moesin (0.001; 0.005)	67.80	3	3.50	119.80
**631**	K1C19_HUMAN	Keratin, type I cytoskeletal 19 (<0.0001; <0.0001)	44.10	2	7.80	142.88
**643**	K1C19_HUMAN	Keratin, type I cytoskeletal 19 (<0.0001; <0.0001)	44.10	9	27.30	527.99
**632**	K1C19_HUMAN	Keratin, type I cytoskeletal 19 (<0.0001; <0.0001)	44.10	11	36.00	856.99
**840**	ANXA3_HUMAN	Annexin A3 (0.0001; 0.0002)	36.40	2	13.90	149.55
**1048**	PCNA_HUMAN	Proliferating cell nuclear antigen (0.016; 0.002)	29.09	13	59.00	97.00
**1171**	MIXTURE 1 DBNL_HUMAN TBA1B_HUMAN	Drebrin-like protein Tubulin alpha-1B chain (0.034; 0.001)	48.46 50.80	17 16	34.00 39.00	88.00 86.00
**1209**	T191A_HUMAN	Transmembrane protein 191A (0.001; 0.014)	18.06	4	33.00	30.00
**1147**	EPHA6_HUMAN	Ephrin type-A receptor 6 (0.0772; <0.0001)	117.901	8	13.00	40
**794**	HSP7C_HUMAN	Heat shock cognate 71 kDa protein (0.026; 0.131)	70.90	3	7.60	214.36

**Table 3:** Pathway enrichment analysis of up-regulated proteins in BRAFV600E mutant HT-29 colon cancer cells using the Reactome Pathway Database.

Pathway Identifier	Pathway Name	#Entities Found	#Entities Total	Entities *p*-Value	Entities FDR	Submitted Entities Found
**R-HSA-9614399**	Regulation of localization of FOXO transcription factors	2	12	1.44 × 10^−4^	0.009	YWHAQ; YWHAZ
**R-HSA-75035**	Chk1/Chk2(Cds1) mediated inactivation of Cyclin B: Cdk1 complex	2	13	1.69 × 10^−4^	0.009	YWHAQ; YWHAZ
**R-HSA-111447**	Activation of BAD and translocation to mitochondria	2	15	2.25 × 10^−4^	0.009	YWHAQ; YWHAZ
**R-HSA-114452**	Activation of BH3-only proteins	2	30	8.88 × 10^−4^	0.026	YWHAQ; YWHAZ
**R-HSA-390522**	Striated Muscle Contraction	2	36	0.001	0.028	TPM1
**R-HSA-445355**	Smooth Muscle Contraction	2	39	0.001	0.028	TPM1
**R-HSA-109606**	Intrinsic Pathway for Apoptosis	2	55	0.003	0.041	YWHAQ; YWHAZ
**R-HSA-5625740**	RHO GTPases activate PKNs	2	63	0.004	0.046	YWHAQ; YWHAZ
**R-HSA-9614085**	FOXO-mediated transcription	2	66	0.004	0.046	YWHAQ; YWHAZ
**R-HSA-1445148**	Translocation of SLC2A4 (GLUT4) to the plasma membrane	2	72	0.005	0.049	YWHAQ; YWHAZ
**R-HSA-69473**	G2/M DNA damage checkpoint	2	78	0.006	0.059	YWHAQ; YWHAZ
**R-HSA-5628897**	TP53 Regulates Metabolic Genes	2	88	0.007	0.059	YWHAQ; YWHAZ
**R-HSA-8869496**	TFAP2A acts as a transcriptional repressor during retinoic acid induced cell differentiation	1	5	0.007	0.059	NPM1
**R-HSA-1237112**	Methionine salvage pathway	1	6	0.009	0.061	ENOPH1
**R-HSA-977347**	Serine biosynthesis	1	9	0.013	0.078	PSPH
**R-HSA-1640170**	Cell Cycle	4	670	0.015	0.078	NPM1; YWHAQ; CAPG; YWHAZ
**R-HSA-3700989**	Transcriptional Regulation by TP53	3	367	0.016	0.078	NPM1; YWHAQ; YWHAZ
**R-HSA-2514853**	Condensation of Prometaphase Chromosomes	1	11	0.016	0.078	CAPG
**R-HSA-9013700**	NOTCH4 Activation and Transmission of Signal to the Nucleus	1	11	0.016	0.078	YWHAZ
**R-HSA-69481**	G2/M Checkpoints	2	151	0.020	0.078	YWHAQ; YWHAZ
**R-HSA-392517**	Rap1 signaling	1	16	0.023	0.078	YWHAZ
**R-HSA-450604**	KSRP (KHSRP) binds and destabilizes mRNA	1	17	0.025	0.078	YWHAZ
**R-HSA-109581**	Apoptosis	2	182	0.029	0.078	YWHAQ; YWHAZ
**R-HSA-6804115**	TP53 regulates transcription of additional cell cycle genes whose exact role in the p53 pathway remain uncertain	1	21	0.030	0.078	NPM1
**R-HSA-397014**	Muscle contraction	2	196	0.033	0.078	TPM1
**R-HSA-166208**	mTORC1-mediated signaling	1	24	0.035	0.078	EIF4E
**R-HSA-429947**	Deadenylation of mRNA	1	25	0.036	0.078	EIF4E
**R-HSA-3214842**	HDMs demethylate histones	1	26	0.037	0.078	ARID5B
**R-HSA-5357801**	Programmed Cell Death	2	217	0.040	0.078	YWHAQ; YWHAZ
**R-HSA-1614635**	Sulfur amino acid metabolism	1	28	0.040	0.078	ENOPH1
**R-HSA-8866652**	Synthesis of active ubiquitin: roles of E1 and E2 enzymes	1	30	0.043	0.078	UCHL3
**R-HSA-8949613**	Cristae formation	1	31	0.045	0.078	IMMT
**R-HSA-9013424**	RHOV GTPase cycle	1	33	0.047	0.078	TPM1

**Table 4:** The immunohistochemistry staining intensity of nucleophosmin in colonic adenocarcinomas differing in BRAF mutational status.

	BRAF mut	BRAF wt	
	N = 7 (33%)	N = 14 (67%)	
Staining intensity	mean	mean	*p*-value
Nuclear	2	1.43	0.009
Cytoplasmic	1.86	0.86	0.005

**Table 5:** Anti-proliferative effect of PLX4032 in BRAF mutant colon cancer cells with acquired resistance to PLX4032 after 2- and 4-h pre-treatment with cytotoxic concentrations of nucleophosmin inhibitor NSC348884 measured by the MTT assay. Shown here are the results from two independent experiments expressed as mean ± standard deviation.

RKOr		NSC348884					
		2 h			4 h		
		-	IC_50_ (2.67 µM)	2 × IC_50_ (5.34 µM)	-	IC_50_ (2.67 µM)	2 × IC_50_ (5.34 µM)
**PLX4032**	IC_50_ (µM)	31.45 ± 1.17	19.75 ± 1.76	11.88 ± 1.34	33.17 ± 0.95	18.55 ± 1.98	<0.01
	LC_50_ (µM)	92.15 ± 13.07	73.63 ± 5.32	73.38 ± 5.96	84.94 ± 0.61	72.98 ± 2.56	59.69 ± 9.97
**HT-29r**		**NSC348884**					
		**2 h**			**4 h**		
		**-**	**IC_50_** **(3.71 µM)**	**2 × IC_50_** **(7.42 µM)**	**-**	**IC_50_** **(3.71 µM)**	**2 × IC_50_** **(7.42 µM)**
**PLX4032**	IC_50_ (µM)	41.10 ± 2.40	32.95 ± 4.15	20.18 ± 1.42	35.19 ± 3.12	25.16 ± 1.17	<0.01
	LC_50_ (µM)	>100	93.98 ± 5.31	84.57 ± 3.28	85.61 ± 8.86	79.14 ± 6.96	46.56 ± 16.13

**Table 6:** Anti-proliferative effect of PLX4032 in BRAF mutant colon cancer cells with acquired resistance to PLX4032 after 6- and 8-h pre-treatment with cytotoxic concentrations of c-Myc transcription inhibitor IZCZ-3 measured by the MTT assay. Shown here are the results from two independent experiments expressed as mean ± standard deviation.

RKOr		IZCZ-3					
		6 h			8 h		
		-	IC_50_ (0.58 µM)	2 × IC_50_ (1.16 µM)	-	IC_50_ (0.58 µM)	2 × IC_50_ (1.16 µM)
**PLX4032**	IC_50_ (µM)	30.22 ± 0.34	8.73 ± 0.01	4.57 ± 0.63	29.71 ± 1.03	7.74 ± 0.66	2.36 ± 1.30
	LC_50_ (µM)	82.89 ± 6.48	71.09 ± 2.92	66.42 ± 1.49	85.88 ± 1.94	77.06 ± 1.47	61.80 ± 12.90
**HT-29r**		**IZCZ-3**					
		**6 h**			**8 h**		
		**-**	**IC_50_** **(0.35 µM)**	**2 × IC_50_** **(0.70 µM)**	**-**	**IC_50_** **(0.35 µM)**	**2 × IC_50_** **(0.70 µM)**
**PLX4032**	IC_50_ (µM)	37.81 ± 4.17	31.76 ± 3.29	26.43 ± 4.51	39.99 ± 1.49	32.03 ± 3.13	17.90 ± 3.23
	LC_50_ (µM)	90.13 ± 1.02	75.49 ± 1.76	77.49 ± 0.52	94.27 ± 9.43	80.13 ± 1.28	73.30 ± 1.00

## Supplementary Materials

The following are available online at https://www.mdpi.com/article/10.3390/ijms22126174/s1.
