# Supplementary material for: Proteomic Profiling of BRAFV600E Mutant Colon Cancer Cells Reveals the Involvement of Nucleophosmin/c-Myc Axis in Modulating the Response and Resistance to BRAF Inhibition by Vemurafenib
Source: Int J Mol Sci. 2021 Jun 8;22(12):6174. doi: 10.3390/ijms22126174 (PMC8228139; doi:10.3390/ijms22126174)
Supplement: Supplementary file 1 [file ijms-22-06174-s001.zip › ijms-1209481-supplementary.pdf]

Supplementary Material

Supplementary Table S1. Gene Ontology analysis of up-regulated proteins ($p < 0.05$) in BRAFV600E mutant HT-29 colon cancer cells based on cellular localization.

**Cellular compartment** ($p < 0.05$)				
**94.12 %**	GO:0044424~ Intracellular part	93.75 %		GO:0043229~intracellular organelle
		75.00 %		GO:0044422~organelle part
		62.50 %		GO:0005634~nucleus
		62.50 %		GO:0005737~cytoplasm
		43.75 %		GO:0005829~cytosol
**41.18%**	Extracellular part			GO:0043230~extracellular organelle
				GO:0070062~extracellular exosome
				GO:1903561~extracellular vesicle
**35.19 %**	GO:0005856~ Cytoskeleton			
**29.41 %**	GO:0030054~ Cell junction	60.00%		GO:0030055~cell-substrate junction
		GO:0005912~ adherens junction	60.00%	GO:0005913~ cell-cell adherens junction
				GO:0005924~cell-substrate adherens junction
		GO:0070161~ anchoring junction		
**17.65 %**	GO:0005925~ Focal adhesion			

Supplementary Table S2. Gene Ontology analysis of up-regulated proteins ($p < 0.05$) in BRAFV600E mutant HT-29 colon cancer cells based on biological processes.

**Biological process** ($p < 0.05$)				
**52.94%**	GO:0006996~ Organelle organization	88.89%		GO:1902589~single-organism organelle organization
		55.56%		GO:0033043~regulation of organelle organization
			83.33%	GO:0010638~positive regulation of organelle organization
**29.41%**	GO:0007010~ Cytoskeleton organization	80.00%		GO:0051493~regulation of cytoskeleton organization
**11.77%**	GO:0061024~ Membrane organization			
**17.65%**	GO:0007006~ Mitochondrial membrane organization	66.67%		GO:1900740~positive regulation of protein insertion into mitochondrial membrane involved in apoptotic signaling pathway
				GO:1901030~positive regulation of mitochondrial outer membrane permeabilization involved in apoptotic signaling pathway
				GO:0097345~mitochondrial outer membrane permeabilization
				GO:1902686~mitochondrial outer membrane permeabilization involved in programmed cell death
				GO:1902110~positive regulation of mitochondrial membrane permeability involved in apoptotic process
**11.77%**	GO:0006605~ Protein targeting			
**17.65%**	GO:0098609~ Cell-cell adhesion	GO:0098641~Cadherin binding involved in cell-cell adhesion		GO:0045296~ Cadherin binding
		GO:0098632~Protein binding involved in cell-cell adhesion		
**29.41%**	GO:0051130~ Positive regulation of cellular component organization			
**58.82%**	GO:0048518~ Positive regulation of biological process			
**17.65%**	GO:0045787~ Positive regulation of cell cycle	GO:0000082~G1/S transition of mitotic cell cycle		
		GO:0044843~cell cycle G1/S phase transition		

**11.77%**	GO:0046605~ Regulation of centrosome cycle		
**23.53%**	GO:0014070~ Response to organic cyclic compound		
**29.41%**	GO:0046907~ Intracellular transport	40.00%	GO:0006405~RNA export from nucleus

Supplementary Table S3. Gene Ontology analysis of up-regulated proteins ($p < 0.05$) in BRAFV600E mutant HT-29 colon cancer cells based on molecular function.

**Molecular Function ($p < 0.05$)**			
**29.41%**	GO:0044822~ Poly(A) RNA binding		
**17.65%**	GO:0019904~ Protein domain specific binding		
**17.65%**	GO:0045296~ Cadherin binding		GO:0098641~ Cadherin binding involved in cell-cell adhesion
**17.65%**	GO:0098632~ Protein binding involved in cell-cell adhesion		

Supplementary Table S4. Top eight significant modules in the PPI network of up-regulated proteins in BRAFV600E mutant HT-29 colon cancer cells identified by Cytoscape plugin MCODE. Hub proteins were ranked in Cytohubba plugin of Cytoscape based on the MCC (maximal clique centrality) and degree (node connect degree) topological analysis methods. Different degrees of colour ranging from red to orange represent the rank of connectivity degree, where red denotes the highest rank.

<table>
<tr><th>CLUSTER (nodes/ edges)</th><th>MCODE score</th><th></th><th></th><th></th><th></th><th></th></tr>
<tr><td rowspan="11">20/176</td><td rowspan="11">18.526</td><td rowspan="11">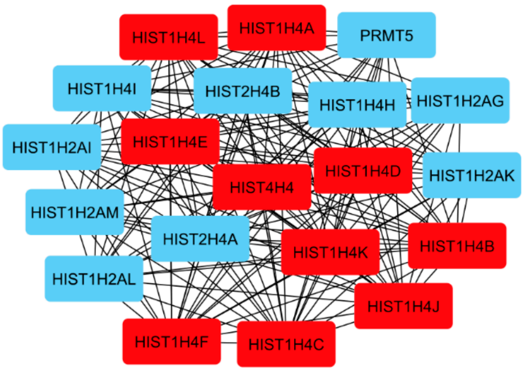
</td><td></td><td>Hub genes</td><td>MCC</td><td>Degree</td></tr>
<tr><td>1.</td><td>HIST4H4</td><td>1.65×10^{12}</td><td>19</td></tr>
<tr><td>2.</td><td>HIST1H4F</td><td>1.65×10^{12}</td><td>19</td></tr>
<tr><td>3.</td><td>HIST1H4L</td><td>1.65×10^{12}</td><td>19</td></tr>
<tr><td>4.</td><td>HIST1H4E</td><td>1.65×10^{12}</td><td>19</td></tr>
<tr><td>5.</td><td>HIST1H4J</td><td>1.65×10^{12}</td><td>19</td></tr>
<tr><td>6.</td><td>HIST1H4C</td><td>1.65×10^{12}</td><td>19</td></tr>
<tr><td>7.</td><td>HIST1H4A</td><td>1.65×10^{12}</td><td>19</td></tr>
<tr><td>8.</td><td>HIST1H4B</td><td>1.65×10^{12}</td><td>19</td></tr>
<tr><td>9.</td><td>HIST1H4D</td><td>1.65×10^{12}</td><td>19</td></tr>
<tr><td>10.</td><td>HIST1H4K</td><td>1.65×10^{12}</td><td>19</td></tr>
<tr><td rowspan="10">15/96</td><td rowspan="10">13.714</td><td rowspan="10">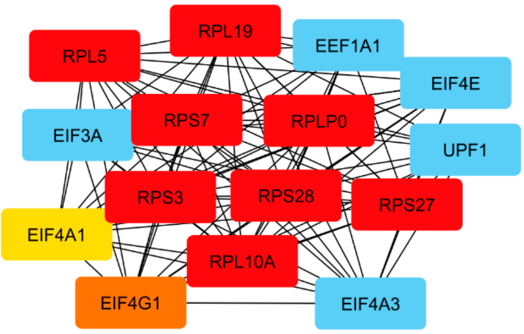
</td><td>1.</td><td>RPS27</td><td>8.38×10^{7}</td><td>14</td></tr>
<tr><td>2.</td><td>RPLP0</td><td>8.38×10^{7}</td><td>14</td></tr>
<tr><td>3.</td><td>RPL5</td><td>8.38×10^{7}</td><td>14</td></tr>
<tr><td>4.</td><td>RPS28</td><td>8.38×10^{7}</td><td>14</td></tr>
<tr><td>5.</td><td>RPS7</td><td>8.38×10^{7}</td><td>14</td></tr>
<tr><td>6.</td><td>RPS3</td><td>8.38×10^{7}</td><td>14</td></tr>
<tr><td>7.</td><td>RPL19</td><td>8.38×10^{7}</td><td>14</td></tr>
<tr><td>8.</td><td>RPL10A</td><td>8.38×10^{7}</td><td>14</td></tr>
<tr><td>9.</td><td>EIF4G1</td><td>8.38×10^{7}</td><td>13</td></tr>
<tr><td>10.</td><td>EIF4A1</td><td>8.38×10^{7}</td><td>13</td></tr>
<tr><td rowspan="3">8/28</td><td rowspan="3">8.000</td><td rowspan="3"></td><td></td><td>Hub genes</td><td>MCC</td><td>Degree</td></tr>
<tr><td>1.</td><td>HSPA8</td><td>5040.0</td><td>7</td></tr>
<tr><td>2.</td><td>HNRNPF</td><td>5040.0</td><td>7</td></tr>
</table>

		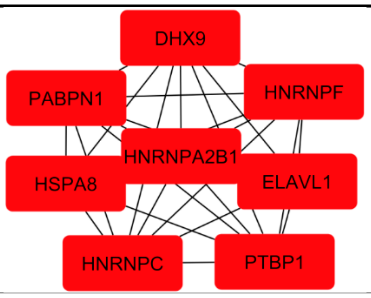	3.	HNRNPA2B1	5040.0	7
			4.	PTBP1	5040.0	7
			5.	ELAVL1	5040.0	7
			6.	PABPN1	5040.0	7
			7.	DHX9	5040.0	7
			8.	HNRNPC	5040.0	7
**19/55**	6.111	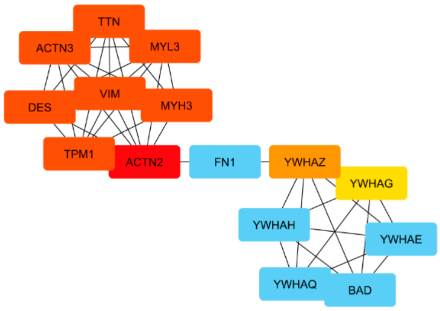	1.	ACTN2	5041.0	8.0
			2.	MYH3	5040.0	7.0
			3.	MYL3	5040.0	7.0
			4.	DES	5040.0	7.0
			5.	VIM	5040.0	7.0
			6.	ACTN3	5040.0	7.0
			7.	TTN	5040.0	7.0
			8.	TPM1	5040.0	7.0
			9.	YWHAZ	121.0	6.0
			10.	YWHAG	120.0	5.0
**5/10**	5.000	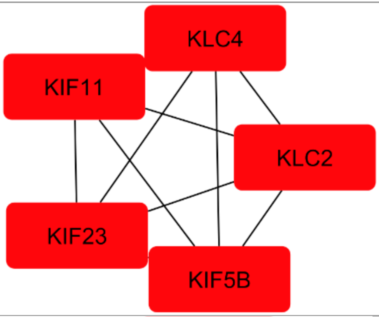	1.	KIF5B	24.0	4
			2.	KLC2	24.0	4
			3.	KIF23	24.0	4
			4.	KIF11	24.0	4
			5.	KLC4	24.0	4
**5/10**	5.000	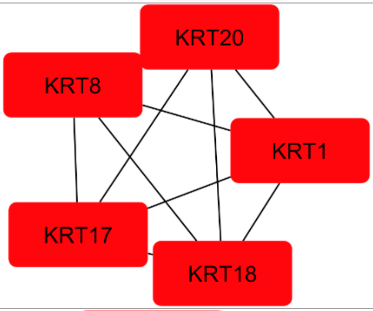	1.	KRT1	24.0	4
			2.	KRT18	24.0	4
			3.	KRT17	24.0	4
			4.	KRT8	24.0	4
			5.	KRT20	24.0	4
**4/6**	4.000	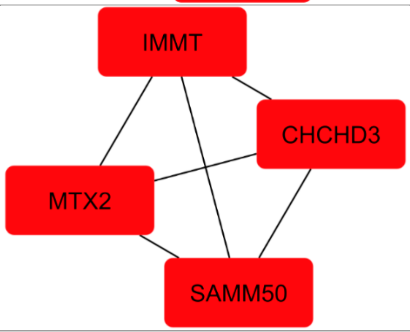	1.	MTX2	6.0	3.0
			2.	IMMT	6.0	3.0
			3.	SAMM50	6.0	3.0
			4.	CHCHD3	6.0	3.0
**5/8**	4.000		1.	HIST1H2BD	8.0	4.0
			2.	H2AFX	8.0	4.0
			3.	ATF2	6.0	3.0
			4.	JUN	6.0	3.0

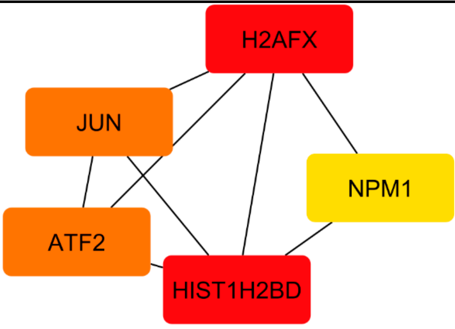	5.	NPM1	2.0	2.0

Supplementary Table S5. Anti-proliferative effects of PLX4032 in BRAF mutant colon cancer cells and the cells with acquired resistance to PLX4032. Shown here are the results from two independent experiments expressed as mean ± standard deviation.

	**HT-29**	**HT-29r**	**RKO**	**RKOr**
IC_{50} (μM)	4.78 ± 0.29	37.39 ± 1.97	3.03 ± 1.03	29.76 ± 0.62
LC_{50} (μM)	71.98 ± 2.28	84.81 ± 4.40	49.62 ± 4.71	74.29 ± 2.28

Supplementary Table S6. The immunohistochemistry staining intensity values for phospho-NPM1 (Thr199) in colonic adenocarcinomas differing in BRAF mutational status. Total scoring is shown for each analyzed tissue sample whereas values in brackets are indicative of nuclear intensity, cytoplasmic intensity and distribution, respectively (mut = mutant; wt= wild type).

**BRAF status**	**KRAS status**	**p-NPM1 (Thr199) staining score**
mut	wt	6 (2+2+2)
mut	wt	6 (2+2+2)
mut	wt	4 (2+1+1)
mut	wt	5 (2+2+1)
mut	wt	6 (2+2+2)
mut	wt	6 (2+2+2)
mut	wt	5 (2+2+1)
wt	mut	5 (2+2+1)
wt	mut	5 (2+2+1)
wt	mut	3 (1+1+1)
wt	mut	2 (1+0+1)
wt	mut	4 (2+1+1)
wt	mut	3 (1+0+2)
wt	mut	3 (1+0+2)
wt	mut	3 (1+1+1)
wt	wt	6 (2+2+2)
wt	wt	3 (1+1+1)
wt	wt	4 (1+1+2)
wt	wt	3 (1+0+2)
wt	wt	4 (2+1+1)
wt	wt	4 (2+0+2)
